# CCL2: An important cytokine in normal and pathological pregnancies: A review

**DOI:** 10.3389/fimmu.2022.1053457

**Published:** 2023-01-06

**Authors:** Zhi Lin, Jia-Lu Shi, Min Chen, Zi-Meng Zheng, Ming-Qing Li, Jun Shao

**Affiliations:** ^1^ Laboratory for Reproductive Immunology, Hospital of Obstetrics and Gynecology, Fudan University, Shanghai, China; ^2^ Department of Gynecology, Hospital of Obstetrics and Gynecology, Fudan University, Shanghai, China; ^3^ National Health Commision (NHC) Key Lab of Reproduction Regulation, Shanghai Institute for Biomedical and Pharmaceutical Technologies, Fudan University, Shanghai, China; ^4^ Shanghai Key Laboratory of Female Reproductive Endocrine Related Diseases, Hospital of Obstetrics and Gynecology, Fudan University, Shanghai, China; ^5^ Department of Obstetrics and Gynecology, Jinshan Hospital of Fudan University, Shanghai, China

**Keywords:** CCL2, CCR2, trophoblast, decidua, abortion, preeclampsia, preterm labor

## Abstract

C-C motif ligand 2 (CCL2), also known as monocytic chemotactic protein 1 (MCP-1), is an integral chemotactic factor which recruits macrophages for the immune response. Together with its receptors (e.g., CCR2, ACKR1, and ACKR2), they exert noticeable influences on various diseases of different systems. At the maternal-fetal interface, CCL2 is detected to be expressed in trophoblasts, decidual tissue, the myometrium, and others. Meanwhile, existing reports have determined a series of physiological regulators of CCL2, which functions in maintaining normal recruitment of immunocytes, tissue remodeling, and angiogenesis. However, abnormal levels of CCL2 have also been reported to be associated with adverse pregnancy outcomes such as spontaneous abortion, preeclampsia and preterm labor. In this review, we concentrate on CCL2 expression at the maternal-fetal interface, as well as its precise regulatory mechanisms and classic signaling pathways, to reveal the multidimensional aspects of CCL2 in pregnancy.

## 1 Introduction

Chemokine C-C motif ligand 2 (CCL2) whose common name is monocytic chemotactic protein 1 (MCP-1), is encoded by the *CCL2* gene which is located on chromosome 17q11.2 (1). Belonging to the CC chemokine superfamily, the inflammatory chemoattractant is made up of 76 amino acids and characterized by four regions of β-sheet that include residues 9–11 (β0), 27–31 (β1), 40–45 (β2), and 51–54 (β3) and two helical regions (2). Besides, like other members of CC chemokine superfamily, it also shares two disulphide bonds at amino acid 34-59 and 35-75 and the conserved C-C motif containing two adjacent cysteines (3). Additionally, the flexible N-terminal region located in the conserved C-C motif is believed to be important in receptor activation (4). CCL2 can be secreted by numerous cell types including endothelial cells, activated monocytes, fibroblasts, vascular smooth muscle cells (VSMCs), renal tubular epithelial cells, astrocytes, microglia, and neurons (5–11). Combination and activation of the seven transmembrane G-protein-coupled receptor C–C chemokine receptor type 2 (CCR2) primarily direct myeloid and lymphoid cell migration, especially blood monocytes, memory T lymphocytes, and natural killer (NK) cells (12). Furthermore, part of CCL2 can also bind to the atypical chemokine receptor 1 (ACKR1), ACKR2, and the glycosaminoglycan chains of proteoglycans including heparan sulfate, heparin, and dermatan sulfate, and then creates a series of reactions (13, 14).

In general, CCL2, especially CCL2 - mediated cell migration, has an emerging role in human pathologic process. On the one hand, it plays a critical role in engendering the adaptive immune response and contributes to the pathogenesis of a variety of diseases such as rheumatoid arthritis. In this process, inflammatory stimuli activate the expression of CCL2 to sustain and aggravate Th17 cell recruitment to the specific location, followed by the production of inflammatory cytokines and other successive responses (14, 15). CCL2 also appears to have a vicious role in the tumor microenvironment (16). Evidence has been provided that the CCL2-CCR2 axis can be stimulated by tumor necrosis factor alpha (TNF-α) from tumors cells in the tumor development to further recruit tumor-associated macrophages(TAMs) who helps cancer cells escape from immune system, and finally prompt the development of tumor. For example, the overexpression of CCL2 enhances the outgrowth, invasion, and metastasis of the 4T1 murine mammary cancer cell line which is one of the most widely used breast cancer models (17–19). Besides, in the nervous system, CCL2, expressed by dorsal root ganglia (DRG) under the influence of sterile alpha and Toll/interleukin-1 receptor motif-containing 1 (Sarm1), will in turn boost the growth potential of DRG through the accumulation of macrophages in the distal nerve segment. Also, CCL2 is found to be implicated in the neuropathic pain (20). In terms of metabolic illnesses, CCL2 appears to participate in tissue damage and insulin resistance in the setting of diabetic nephropathy (21). Research also shows that CCL2 deficiency in diabetic (*db/db*) mice which is a recognized model of type 2 diabetes with a mutation of the diabetes (db) gene encoding for the leptin receptor reduces renal macrophage accumulation and the progression of diabetic renal injury (22). Furthermore, several studies have demonstrated that endothelial cells on arterial vessels can release CCL2 to upregulate the cell adhesion molecules like vascular cell adhesion molecule 1 (VCAM-1), intercellular adhesion molecule 1 (ICAM-1), P-selectin, and E-selectin, to trigger cell arrest and facilitate leukocyte immigration into atherosclerotic lesions (23). This dysfunction is also strongly correlated with hypertension and other cardiovascular diseases (24, 25).

Previous evidence has indicated that CCL2 is secreted by human first-trimester decidual tissue in an autocrine manner through the extracellular signal-regulated kinases (ERK)/mitogen-activated protein kinase (MAPK) pathway and is regulated by pregnancy-associated factors (26), and the difference in its concentration can lead to both normal pregnancy progression and pathological pregnancy. In this review, we critically summary the expression of CCL2 at the maternal-fetal interface and the significance of CCL2 in normal and pathological pregnancies to untangle the connections.

## 2 The expression of CCL2 at the maternal-fetal interface.

### 2.1 CCL2 in the trophoblast cells

Trophoblast cells can be functionally divided into villous cytotrophoblast (vCTB), syncytiotrophoblast (STB), and extravillous trophoblasts (EVT). It is well acknowledged that vCTBs and EVTs can produce moderate amounts of CCL2 in early gestation (27, 28). According to Naruse’s investigation, the level of CCL2 in 8-10 weeks of gestation did not differ from the level in 12–14 weeks but the significance of this result has not been clarified (29). Although there are few literatures regarding CCL2 in STBs, some reports about CCR2 such as ACKR2 which is vital in STB should be given more attention. The reason for its atypia is that ACKR2 is parallel in structure and bonding capacity with its ligands to typical chemokines but plays a different part in inflammatory and immune regulation (30). More specifically, being present in early endosomes of STB, ACKR2 mainly internalizes and eliminates redundant CCL2 to deter unnecessary cell transport (31) (**Table 1**).

**Table 1:** The expression of CCL2/CCR2 at the maternal-fetal interface.

	CCL2	CCR2	ACKR2
**villous cytotrophoblast**	**+**	**+**	**-**
**Syncytiotrophoblast**	**-**	**-**	**+**
**extra-villous trophoblast**	**+**	**-**	**-**
**Decidual stromal cells**	**+**	**+**	**-**
**Decidual macrophages**	**+**	**+**	**-**
**CD4^+^T cells**	**-**	**+**	**-**
**Decidual nature killer cells**	**-**	**-**	**-**

### 2.2 CCL2 in the decidual stromal cells

In many studies, immunohistochemistry and ELISA have detected the strong expression and secretion of CCL2 in decidual stromal cells (DSC) from normal pregnant women ending up their gestation for nonmedical reasons in the first trimester (32–34). Specifically, after primary culture *in vitro*, the transcription and autocrine secretion of CCL2 in 72-h supernatant liquid is confirmed to be positively correlated with time (35). He et al. gained similar outcomes using reverse transcription-polymerase chain reaction (RT-PCR) while CCR2 was also found to be abundantly expressed in the cytoplasm and on the cellular membrane of DSC through Immunohistochemical and Immunocytochemical staining. However, in the same study, immunocytochemical characterization presented more pigmentation of CCR2 in endometrium stromal cells (ESC) than in DSCs, suggesting the certain function of CCR2 in establishing and sustaining the relationship between DSCs and ESCs (26).

### 2.3 CCL2 in the decidual immune cells

Broadly speaking, decidual immune cells (DICs) at the maternal-fetal interface include antigen-presenting cells (APCs), T cells and NK cells (36). Macrophages, serving as APCs, are the most closely associated with both CCL2 and CCR2 among DICs. Decidual macrophages discharge similar amounts of CCL2 as peripheral derived macrophages to recruit blood macrophages into the decidua (37). CCR2**^+^** CD11c^high^ macrophages, one of the subtypes of decidual macrophages in early pregnancy, can be gathered to EVT through combining with CCL2 and excessively express relevant genes such as interleukin-1 beta (IL-1β) and prostaglandin G/H synthase 2 (PTGS2/COX2) in order to establish a proinflammatory status for the phagocytosis of pathogens (38). As the least numerous DICs in decidua, T cells are also the nonnegligible target of CCL2 (39). The expression of CCR2 by CD4**^+^** T cells is much higher in the decidual tissue than in peripheral blood (40). Its subgroups, including T helper (Th)1, Th2, Th17, and T regulatory cells (Treg cells), collaborate with CCL2 to realize their physiologic properties (41). The NK cells at the maternal-fetal interface are collectively known as uterine natural killer (uNK) cells, which consist of decidual natural killer (dNK) cells and endometrial NK (eNK) cells. And dNK cells account for the largest part of DICs (42). Regrettably, little evidence has illustrated the spontaneous expression of CCL2 in dNK cells. Gibson et al. found that uNK cells from decidua could release CCL2 after the stimulation of estradiol (E2) to modulate vascular function but the exact type of uNK cells (dNK or eNK cells) was unknown (43). Of note, the interaction between CCL2 and CCR2 can assist DICs in regulating the maternal-fetal interface immune microenvironment to promote pregnancy progression (34, 44).

## 3 Regulation of CCL2 expression at the meternal-fetal interface

As described above, CCL2 is of great importance at the maternal-fetal interface and its expression and secretion can be regulated by multiple endogenous factors to assure a friendly uterine microenvironment (**Table 2**). Here, the pathological regulators of pregnancy diseases will be enumerated.

**Table 2:** Regulation of CCL2 expression at the maternal-fetal interface.

Classification	Regulatory factor	Function	Reference
**Hormones**	E2	Boosts the production of CCL2 in DSCs by working on the binding sites for AP-1 and NF-kB	(26, 45)
		Reduces the level of CCL2 in the placenta to control inflammation *via* ERΑ36/TLR4 pathways	(46)
	HCG	Elevates the level of CCL2 protein and mRNA	(47)
	Progesterone	Elevates the level of CCL2 protein and mRNA	(47)
	PGF2Α	Increases CCL2 in a dose-dependent manner involving PLC/PKC, ERK1/2, MAPK p38 and PI3K pathway	(48)
	VIP	Accelerates the expression of CCL2 to be one of decidualization markers	(49, 50)
**Cytokines**	IL-33	Raises the concentration of CCL2 and CCR2 in DSCs through the phosphorylation of NF-kB p65 and ERK1/2	(51)
	RANKL	Enhance CCL2/CCR2 axis concerning with NF-kB pathway	(52)
	TNF -α	Causes the ascent of CCL2 in first trimester trophoblast cells through the activation of MAPK and c-JNK signaling	(53)
	IL-1β	Triggers high expression of CCL2	(54)
**Enzymes and Metabolites**	Thrombin	Augments CCL2 protein expression through PAR-1 mediated pathways including PAR-1/Raf-1/MEK/MAPK cascade responses, PAR-1/Rho/Rho-kinase pathway or non-PAR-1 pathway including PLC-InsP3/Ca^2+^-PKC and downstream ERK1/2 but CCL2 mRNA doesn’t be affected	(55–58)
	HO-1	Advances CCL2 and CCR2 expression in decidual cells	(59)
	LPA	Works on LPA1 receptor of human first-trimester trophoblast cells and then releases CCL2 *via* Gi protein, ERK, PKC, p38, Akt, JNK and NF-kB signaling	(60, 61)
	Lactate	Restrains the expression of CCL2 *via* GPR81	(62)

### 3.1 Hormones

Pregnancy is a complex process in which the concentrations of estrogen, progesterone, and other relevant hormones are much higher than those at any other stages of life (63). We can therefore consider the association between the regulatory role of pregnancy-associated hormones and CCL2. When a woman is pregnant, the estrogen in her state not only takes part in the decidualization and remodeling of uterine tissue, but also boosts the production of CCL2 from DSCs possibly through working on the promoter region containing binding sites for activator protein-1 (AP-1) and nuclear factor kappa B (NF-kB) (26, 45). Another interesting study reported that E2 reduced the level of CCL2 in the placenta to control inflammation and further treat preeclampsia (PE) *via* estrogen receptor Α36 (ERΑ36)-induced toll-like receptor 4 (TLR4) pathways (46). Apart from this, the level of CCL2 mRNA and protein can be drastically elevated by human chorionic gonadotropin (hCG) as well as progesterone but the concrete mechanisms need further exploration (64). Since gestation is actually a mild inflammatory process accompanied by the infiltration of leukocytes and the generation of CCL2 and other inflammatory chemokines (47), pro-inflammatory hormones are expected to regulate the expression of CCL2. Take prostaglandin F2Α (PGF2Α) as an example, it links to PGF2Α receptor (PTGFR) to increase CCL2 in a dose-dependent manner *in vitro* and knockdown of PTGFR reverses the up-regulation. Chen et al. have put forward its signaling pathways in human uterine smooth muscle cells (HUSMCs) in the third trimetster, including phospholipase C/protein kinase C (PLC/PKC), ERK1/2, MAPK p38, and phosphatidylinositol-4,5-bisphosphate 3-kinase (PI3K) signaling (48). Little, however, is known about the pathways triggered by PGF2Α in decidual cells. In addition, vasoactive intestinal peptide (VIP) which originates from the STB in the early trimester and spreads in the decidual stroma can lead the transmission of peripheral monocytes to the decidua *via* accelerating the expression of CCL2 and CCL3 (49). Meanwhile, CCL2 can be regarded as one of the decidualization markers induced by VIP (50).

### 3.2 Cytokines

There is no doubt that cytokines are indispensable in creating an environment more suitable for pregnancy and the interactive network among them is so complicated that plenty of modulatory outcomes can be observed, including cell migration, invasion, and gene expression (65, 66). The adjustment of CCL2 always takes place at the maternal-fetal interface during different trimesters with the help of interleukin (IL)-33, IL-1β, TNF-α, receptor-activator of NF-kB ligand (TNFSF11, also known as RANKL) and so on. In Hu et al.’s investigation, IL-33 treatment raised the concentration of CCL2 and CCR2 in DSCs, and IL-33 inhibitor prevented the change. Phosphorylation of NF-kB p65 and ERK1/2 has also been involved in the above up-regulation process that is beneficial to the proliferation of DSCs and the sustainment of a normal pregnancy (51). Membrane RANKL and RANK proteins are located in DSCs of the first trimester and encourage the growth of DSCs by enhancing the CCL2/CCR2 axis (52). Additionally, Renaud et al. reported that the inducement of TNF-α caused the ascent of CCL2 and CCL5 in trophoblast cells in the first trimester through the activation of MAPK and c-Jun N-terminal kinase (c-JNK) (53). Cumulative evidence supports that when TNF-α is overexpressed, it has an enormous influence on the adverse consequences of pregnancy which will be mentioned below (67, 68). Moreover, *in vitro* experiments conducted by Lockwood et al. showed that decidual cells dealt with IL-1β triggered high expression of CCL2 (54). In short, different cytokines sometimes mediate similar pathways to regulate CCL2 but lead to different impacts, which drives the need for more studies exploring their independent but correlative characteristics in pregnancy.

### 3.3 Enzymes and metabolites

Normal activity of enzymes and metabolism is of major significance for hemodynamics at the maternal-fetal interface. Deep embedment of trophoblast cells into the maternal arteries can reshape vessels with high flow and capacity to ensure adequate nutrition for the fetus (69). Successive penetration of circulating VII complexes results in the synthesis of thrombin in the stromal tissue. In addition to its coagulation function, its moderate level maintains gestational normality and keeps the level of some chemokines including CCL2 and IL-8 under control. If this normal range is exceeded, preeclampsia and recurrent miscarriage will arise more easily (55, 70). Matta et al. found that due to the posttranslational effect, higher concentration of thrombin advanced more CCL2 protein expression in decidual cells of the early stage, but the level of CCL2 mRNA remained unaffected (56). Focusing on the mechanisms, Kawano pointed out that proteinase-activated receptors-1 (PAR-1) was the crucial receptor to regulate thrombin-dependent pathways. When ESC prepared for implantation, Raf-1 got activated after the combination with PAR-1, initiated the mitogen-activated protein kinase kinase (MEK)/ERK cascade responses and finally augmented CCL2 expression (57). Meanwhile, recent findings referred to the PAR-1/Rho/Rho-kinase pathway in EVTs as a necessity of the increase of CCL2 (55). The non-PAR-1 pathway includes PLC-inositol 1,4,5-trisphosphate (InsP3)/Ca2+-PKC, and downstream ERK1/2 (57, 58). Heme oxygenase-1 (HO-1) turns out to be involved in placental angiogenesis, antioxidative surroundings, and macrophage transfer. Absence of HO-1 in pregnant uteri can substantially lessen CCL2 and CCR2 in decidual cells so that monocyte/macrophage infiltration will not be sufficient to maintain a successful pregnancy (59). When it comes to metabolites, lysophosphatidic acid (LPA) from the metabolism of lysophospholipase D increases with advancing gestational weeks. It works on LPA1 receptor of human first-trimester trophoblast cells and then releases CCL2 and IL-8 *via* Gi protein, ERK, PKC, p38, Akt, JNK, and NF-kB signing (60, 61). Conversely, lactate is one of the vital metabolites of violent myometrium contraction and assists in balancing inflammation during delivery. It functions *via* G protein-coupled receptor GPR81 to restrain the overexpression of CCL2 and attenuate the ensued preterm birth (62).

## 4 Biological function of CCL2 at the maternal-fetal interface

**Figure 1:**
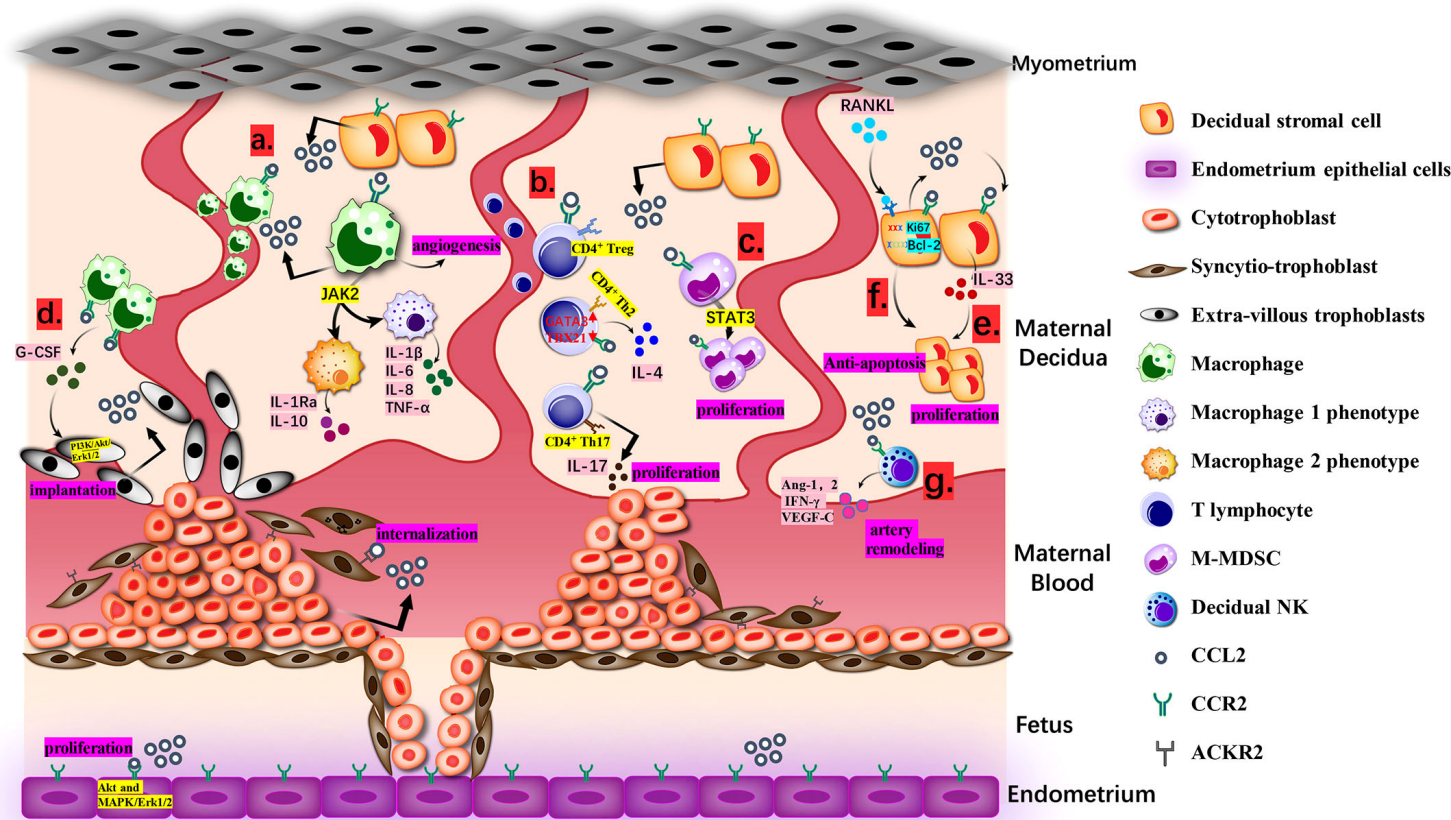
Biological Function of CCL2 at the Maternal-fetal interface. **(A)**. Macrophages from maternal blood produce CCL2 and recruit extra macrophages. Janus kinase 2 (JAK2) is the desirable effector downstream of CCL2-CCR2 for keeping an optional balance of M1 and M2 phenotypes. M1 produces IL-1β, IL-6, IL-8, TNF-α and M2 produces IL-1Ra and IL-10. **(B)**. CCL2 recruits CD4^+^ T cells, including Th 2, Th17 and T regulatory cells (Treg cells). The ration of transcription factors GATA3 and T-bet (TBX21) in naive T cells goes up, deciding the bias of Th2 polarization for the tolerate gestation with accumulative IL-4.Th17 cells approach DSC with the guidance of CCL2-CCR2 axis and then generate IL-17 to endorse trophoblast proliferation and control its apoptosis. **(C)**. M-MDSCs multiply at maternal-fetal interface *via* CCL2/STAT3 pathway and can attenuate the activity of T cells. **(D)**. CCR2^+^CD11c^high^ macrophages can be gathered to EVT *via* combining with CCL2 and then regulate the growth and invasion of EVT through PI3K/Akt/Erk1/2 pathway for stronger implantation. **(E)**. CCL2 causes the proliferation and invasion of DSCs directed by IL-33. **(F)**. CCL2 and CCR2 expression caused by RANKL/RANK increased Ki67 and Bcl-2 and reduced FasL, making sure the anti-apoptosis and increment of DSC. **(G)**. With high level of CCL2, dNK cells generate angiopoietin-1 (Ang-1), Ang-2, IFN-γ and VEGF-C to dedifferentiate and degrade VSMC, which is a requisite step in spiral artery remodeling.

Undoubtedly, under the control of modulators, CCL2 will achieve a dynamic balance and make full use of itself throughout entire proceeding, directly and indirectly (**Figure 1**).

### 4.1 Role in recruitment and regulation of immune cells

CCL2 always enjoys a great reputation for its role in attracting appropriate immune cells to certain tissues, and DICs in pregnancy are not exempt (71). Macrophages, T cells, basophils, mast cells, and NK cells can respond to CCL2 (72). They stem from the mother’s peripheral blood, assemble at the maternal-fetal interface, and then transform into DICs which perform essential functions to guarantee the immune microenvironment and immune tolerance (73, 74).

CCL2 is the most available chemotactic factor to decidual macrophages. After recruitment, decidual macrophages subsequently produce a wide range of inflammatory mediators including CCL2 and attract more macrophages to create a positive feedback (75). In the first 6–12 weeks, CD14**^+^** decidual macrophages are sorted into three types: CCR2**^+^** CD11c^high^, CCR2**^-^** CD11c^high^, and CCR2**^-^** CD11c^low^. With the progression of pregnancy, their respective proportion and cytokine patterns will change and turn into two phenotypes like traditional M1/M2 (76). *In vivo* tests indicated that Janus kinase 2 (JAK2) was the desirable effector downstream of CCL2-CCR2 for keeping an optimal balance of macrophage immune state in the first trimester (34). IL-1β, IL-6, IL-8, and TNF-α from M1-accociated phenotype are directed at pathogenic and inflammatory matters while a considerable amount of IL-1 receptor antagonist (IL-1Ra) and IL-10 from the other one counter undue reactions, and ultimately it achieves the coexistence of the protection from jeopardy and the tolerance of immunity (77).

As for T lymphocytes, CCL2 mostly recruits CD4**^+^** T cells. The shift of CD4^+^CD25^high^ Tregs is guided by CCL-CCR2 axis (78). Huang showed that the number of migrant Tregs was diminished by CCR2 antagonist (64). Furthermore, Th17 cells can be attracted to decidua, partly due to the connection between the expression of CCR2 in Th17 and the expression of CCL2 in DSCs (32, 79). However, Th2 is predominant at the maternal-fetal interface, which is also related to CCL2 (80). With the constant stimulation of CCL2 in the early trimester, the proportion of transcription factors GATA3 and T-bet/TBX21 in naive T cells goes up. And then it leads to the bias of Th2 polarization, followed by an increase in IL-4 and a relative decrease in interferon gamma (IFN-γ), finally contributing to the toleration of gestation (35, 40).

There are other unconventional DICs receiving the regulations of CCL2. Myelomonocytic myeloid-derived suppressor cells (M-MDSCs), characterized as CD14**^+^** HLA-DR**^-/low^** cells, were found to multiply at the maternal-fetal interface *via* the CCL2/transducer and activator of transcription 3 (STAT3) pathway. Their ability to attenuate the activity of T cell has drawn much attention recently and they may also be associated with the status of immunologic unresponsiveness in pregnancy (81). Apart from the proliferation of M-MDSCs, CCL2 can affect the differentiation of dendritic cells (DCs) that develop from CD14**^+^** monocytes and are equipped with the dual ability to present antigens and inhibit immune responses (36, 82). Current supporting evidence is that partially owing to the presence of CCL2, DCs in the first trimester are typically divided into a majority of premature DC-SIGN**^+^** DCs and a minority of CD83**^+^** DCs. After the neutralization of CCL2, the amount of DC-SIGN**^+^** DCs decreased (83). However, according to Jimenez et al., CCL2 appeared to motivate the maturation of DCs (84). This disagreement will not be solved until the comprehensive explanation is proposed.

Interestingly, IL-15, TGF-β, and CXCL12 are engaged in the transition of peripheral CD56^bright^ CD16**^-^** NK cells into dNK cells *via* CD9, CD49a, CD103, CXCR3, and CXCR4 (85). Though CCL2 is not found to participate in the transition described above, it has been reported to mediate the regulatory signal pathway between dNK cells and peripheral blood NK cells (86). Otherwise, CCL2 from DSCs is likely to impair the cytotoxicity of NK cells for maternal-fetal tolerance. The up-regulation of Suppressor of Cytokine Signaling 3 (SOCS3) mediated by CCL2 may result in the inhibition of perforin in NK cells by undermining the activity of STAT family members, especially STAT3 and STAT5 (87).

### 4.2 Contribution to tissue remodeling and embryo implantation

There is broad recognition that at the beginning of pregnancy some necessary alterations in the uterus will happen to achieve perfect endometrial receptivity (88, 89). In humans, distinct from other mammals such as mice, the remodeling of the endometrium is driven by ovarian hormones and a series of chemokines to make provision for embryo implantation (90). During this process, endometrial stromal fibroblasts are specifically converted into decidual cells and have been proved to express CCL2 protein (91). It is likely that CCL2 can mildly strengthen the ability of endometrial epithelial cells to migrate by curbing the transcription of tight junction protein 1 (TJP-1). This modification has only been verified in pigs and remains unknown in humans (92). CCL2 also has a positive influence on the proliferation and vigor of ESC through Akt and MAPK/ERK1/2 rather than MAPK p38 and the JNK signaling pathway, which is advantageous for decidualization (93, 94).

Once trophoblast cells build firm bonds with the prepared endometrium, regional epithelial apoptosis enables trophoblast to intrude into deeper decidua along with the differentiation of trophoblast cells (95, 96). It is CCL2 that produced by DSCs to attract EVTs *via* CCR2 and then the invasiveness is notably heightened (97). In fact, the indirect effect of CCL2 on trophoblast cells is far from negligible. CCL2 attracts macrophages expressing G-CSF to regulate the growth and invasion of EVT through the PI3K/AKT/ERK1/2 pathway for deeper implantation (98). Th17 cells approach DSC with the guidance of the CCL2-CCR2 axis and then generate IL-17 to endorse trophoblast proliferation and control the apoptosis (32). Other secondary assistances can also be offered by CCL2 such as the suppression of cyclooxygenase-2 (COX-2) related to oxidative stress and these make a pregnancy more likely to succeed (99).

### 4.3 Effect on proliferation and invasion of DSCs

DSCs are considered as a major component following the decidualization to back up embryonic growth on the aspects of nutrition, immune tolerance, and anti-inflammation (100, 101). However, the realization of competence relies on natural proliferation and invasion of DSCs, whose relevance to CCL2 has been mentioned in Hu’s study. Neutralizing antibodies to CCL2 reduced the proliferation and invasion of DSCs directed by IL-33, which means CCL2 may play a coordinating role with IL-33 to help DSCs to growth and invade (51). On the other hand, Meng et al. found that the expression of CCL2 and CCR2 caused by RANKL/RANK increased Ki67 and Bcl-2 and reduced FasL, ensuring the anti-apoptosis and increment of DSC. The mechanism behind the condition has not yet been completely understood (52).

Taken together, current researches make it clear that CCL2 is involved more in the interplay between DSCs and DICs than the development of DSCs itself (102). Therefore, future exploration on the latter can be taken into account as one possible orientation for CCL2.

### 4.4 Ability of spiral arteries remodeling

As pregnancy progresses, angiogenesis leads to the generation of expanding spiral arteries that replace existing high resistance ones. It requires the cooperation of EVT, VSMC, endothelial cells, and DICs realized by a succession of angiogenic factors and signaling circuits, including CCL2 (103–106). In the early stage of angiogenesis, dNK cells and macrophages intrude into the wall of spiral arteries and generate angiopoietin-1 (Ang-1), Ang-2, IFN-γ, and VEGF-C to dedifferentiate and degrade VSMC, which is a requisite step in spiral artery remodeling (107, 108). Meanwhile, CCL2 in decidual macrophages assists the appropriate transformation of vessels *via* rationalizing the M1/M2 ratio (109). Though preceding conclusions have demonstrated that macrophages affected the release of proangiogenic factors through the expression of tyrosine kinase *via* immunoglobulin-like and EGF-like domains (TIE2), neuropilin 1 (NRP1) or the transcription of E26 transformation-specific-1 (Ets-1) in endothelial cells of different tissues, we hypothesize whether these proteins fit in macrophages at the maternal-fetal interface depends on different characteristics in separate vascular beds (110–113). In the second and third trimester, further increase in spiral blood flow implies the persistence and improvement of this process. Ma et al. observed that placental tissue cultivated *in vitro*, to some degree, steered proliferation, migration, adhesion, invasion, and tube formation of HUVEC. As one of the representative elements, high level of CCL2 disintegrated the extracellular matrix (ECM) *via* increasing the secretion of MMP-1 and this propelled the expression of fibroblast growth factor, platelet-derived growth factors, and vascular endothelial growth factor to elicit signal pathways in HUVEC, including MAPKs (114, 115). However, *in vivo* mechanisms demand more careful verification. All in all, the role of CCL2 in other steps of the development of uteroplacental circulation deserves to be elucidated.

## 5 The role of CCL2 in pathological pregnancy

When the level of CCL2 moves out of the normal physiological range, its functions introduced above will be ineffective and cause several diseases of pregnancy (116). More and more researchers have monitored changes in CCL2 during the development of different pathological conditions, suggesting that it could be useful for prediction and treatment.

### 5.1 Spontaneous abortion

Spontaneous abortion (SA), or miscarriage, is the most frequent cause of autogenic pregnancy demise before the 24^th^ gestational week (117). Chromosomal abnormalities is the principal menace and aberrant level of chemokines is regarded as an additional hazard (118). For example, the rise of TNF-α and macrophage inflammatory protein 1-alpha (MIP-1a) in women who spontaneously abort is apparent, while CCL2 is a disputed point (119). Zhang et al. found that the amount of CCL2 mRNA in chorionic and decidual tissues of an SA cohort surpassed that of the control (120). Later, another study from Hannan et al., examining the plasma of miscarriage and control groups, did not witness any expression discrepancy of CCL2, CCL5, CCL7, and C-X3-C Motif Chemokine Ligand 1 between the two groups (121). Different methodology and compositions in decidua and plasma may be the reason behind it. However, when it comes to recurrent pregnancy loss (RPL; covering three or more unsuccessful pregnancies), recent data and literatures have reached agreement that respondents with RPL expressed higher level of CCL2 than normal gravidas (122).

In fact, there is a more inflamed microenvironment in SA patients that is identified to be consistent with the growing tendency of pro-inflammatory factors. TLR4 is assigned to the Toll-like receptors family and can activate the NF-kB pathway (123). Under circumstances of uterine immune imbalance in RPL patients, exorbitant TLR4 tends to mediate the alteration of T cells to Th1 and the generation of Th1 cytokines like TNF-α and INF-γ *via* CCL2/CCR2, which remains to be further elucidated (35). TNF-α can accordingly prompt the expression of CCL2 to create a vicious circle (86, 124). With higher level of CCL2, more M1 macrophages will be recruited and stimulated to secret more pro-inflammatory factors that enforce CCL2 expression to create a positive feedback loop (125, 126). In addition, *in vivo* excessive IL-1β resulting from the decrease in IL-1Ra level during the window of implantation multiplies the level of CCL2 mRNA and protein (127). The immune variations on account of the cell dysfunction are likely to contribute to the pathogenesis of abortion but the underlying mechanisms need to be clarified.

### 5.2 Preeclampsia

Preeclampsia (PE) is an intricate pregnancy complication that presents with newly developed hypertension after the 20^th^ gestational week and causes placental dysfunction and maternal organ abnormalities (128). It is broadly accepted that impaired intrusion of trophoblasts breaks stable angiogenesis, leading to endothelial malfunction, oxidative stress, and improper inflammation (129). The variation and pertinent regulators of CCL2 in this process are worth consideration. Evidence in PE patients has suggested that the concentration of CCL2 was well beyond the normal range, both in the plasma and placenta (130). It was conformed to the results of Cui’s investigation that CCL2 expression was higher in patients during mid-pregnancy and increased with the expansion of severity (131). On the contrary, a cohort study targeting the level of CCL2 in maternal circulation during the first trimester to forecast the incidence of PE showed that patients with PE produced less CCL2 in early pregnancy than control patients. Therefore, CCL2 can be a reliable biomarker for predicting PE (132). Furthermore, Yan et al. shed light on the pathological regulation and suggested that hypoxia in PE lowered the expression of ACKR2 (D6), as well as impelled the upregulation of CCL2 by negative feedback and the apoptosis of trophoblasts (30, 133). Zhang et al. made use of nuclear factor erythroid 2-related factor 2 (Nrf-2) inhibitor *in vivo* to confirm that low levels of Nrf-2, which is relevant to reactive oxygen species (ROS), could increase CCL2 in placental tissue (134). Li et al. proposed the original concept that cell‐free fetal DNA (cffDNA) from dead STB or CTB accrued in patients with PE. Melanoma 2 (AIM2) as a DNA sensor in trophoblasts distinguished high level of cffDNA and excited the overexpression of CCL2 (135).

Additionally, there are other studies emphasizing the influence of changing levels of CCL2 on PE. In one of the monocyte/macrophage-specific discussions, partly because of the increase of CCL2, the number of CD14**^+^** CD11c**^+^** CD163**^-^** monocytes markedly grew with the suppression of CD14**^+^** CD11c**^+^** CD163**^+^** monocytes (136). The former enhanced Fas-intermediated apoptosis of EVT to disturb intact placental implantation (137). On the other hand, incremental CCL2 and IL-8 attracted circulating monocytes to vessel walls and to harm the vascular endothelium in a way similar to that of atherosclerotic lesions. In this regard, Scott substantiated that antioxidation treatment with vitamin E *in vivo* and *in vitro* both intercepted the production of CCL2 *via* the TLR-NF-kB signal, mitigating the negative influence (46, 138, 139). As for systematic inflammatory disorders, it is also associated with the overexpression of CCL2, especially in the last trimester (140).

Overall, the above-mentioned interpretations demonstrate the value of CCL2 in pathogenesis and outcomes of PE, but further investigation into the biomolecular pathway is needed.

### 5.3 Preterm labor

Preterm labor (PTL) is considered as birth before completed 37 weeks of gestation (141). In fact, the pathophysiology of it is similar to the term labor. Generally speaking, in the last trimester, more leukocytes are attracted into the myometrium to form an “inflammatory microenvironment” with the final onset of parturition. However, this process sometimes happens ahead of schedule because of several pathologic processes and the impaired immune tolerance, and PTL occurs (142, 143). Inflammation arising from intra-amniotic infection (IAI) is universally recognized to have a certified causal relationship with preterm delivery. It is featured with anomalous infiltration of monocytes/macrophages and neutrophils, followed by the increasing level of immune mediators and pro-inflammatory cytokines (144). CCL2, one of them, has turned out to be excessive no matter in tissue of preterm pregnancies or models of PTL. Phetcharawan et al. obtained placental samples from pregnancies delivered between 25.3–36.0 weeks and discovered that compared with PTL alone, placental CCL2 mRNA level in PTL with IAI was higher, which demonstrated the essential role of CCL2 in PTL caused by IAI (145, 146). Also, lipopolysaccharide (LPS), a bacterial product, can be administrated to pregnant animals to imitate infection and establish the model of PLT. In addition to placental tissues from models, uterine tissues can embody the difference of CCL2 expression. For example, Marcia et al. adopted it in the *in vivo* test and got the result that myometrial SMCs of the uterine tissues from the LPS-treated group produced more CCL2 than the control ones (147). There are a few of upstream signal molecules regulating the expression of CCL2, some of which have been regarded as targets for studies on treatment and prevention. The interferon regulatory factor 5 (IRF5) was found elevated in myometrial cells to respond to inflammation. *In vitro* siTRF5 experiment found the diminishment of TNF-mediated CCL2 mRNA expression, corroborating that IRF5 enhanced CCL2 expression at the transcriptional level. Concretely, IRF5 combined with RELA subunit of NF-ΚB activated by TNF-α and stimulated NF-ΚB signal pathway to secret more CCL2 (148). GPRs belong to another family related closely to labor and GPR91 is one of them who are active in inflammation during PTL. The knockdown of GPR91 had effect on the reduction of IL1β-mediated rather than TNF-mediated CCL2 mRNA expression and secretion, which might attribute to their recruitment of different messengers to regulate downstream genes (149). Other experiments focusing on the therapy of PTL mainly acted on different targets but ended up with NF-kB signal pathway to decrease the secretion of CCL2 (150–152). Therefore, CCL2 seems to make a difference in terminal inflammatory pathway and it is worth intensive studying how CCL2 exerts its function (153).

## 6 Conclusion

In conclusion, a growing body of evidence demonstrates that CCL2 was produced at the maternal-fetal interface, aided by pregnancy-associated regulatory factors, especially in decidual stromal cells. As a valuable chemoattractant, it fosters the migration of a different sort of decidual immune cell from peripheral tissue to the decidua by binding typical or atypical receptors. Meanwhile, equally important is its contribution to the decidualization of the endometrium, invasion of trophoblasts, and proliferation of decidual stromal cells. Its participation in the NF-ĸB pathway, Akt signaling pathway, and ERK pathway also bridges the communication between cells. However, when exogenous infections or endogenous changes disorder the level of CCL2, impaired cell function and altered immunological tolerance will appear, leading to miscarriage, preeclampsia or preterm labor (**Table 3**). And currently, some recent experimental works have chosen deviant molecules upstream of the CCL2-CCR2 axis as therapeutic targets to normalize CCL2 expression and improve disadvantageous outcomes. Accordingly, through this literature summary, we can try to realize the clinical function of CCL2 as possible and improve the pathological pregnancy outcome by more precise regulation of CCL2 level in the future. Hopefully, our growing knowledge of new methodologies such as organoid culture models, CRISPR technology, and mesenchymal stem cells can establish a more spacious platform for research about CCL2 in normal and pathological pregnancies (154–156) to meet the challenges attributing to differences between species and complex crosstalk happening *in vivo*.

**Table 3:** The behaviors of CCL2 in different pregnancy outcomes at specific trimesters .

Normal pregnancy	Abortion	Pre-eclampsia	Preterm labor
Attracts macrophages to keep a balanced immune state in the first trimester (34)	Recruits more M1 macrophages to secret more pro-inflammatory factors in the early trimester (125, 126)	Enhances Fas-intermediated apoptosis of EVT in the second trimester (137)	Increases the infiltration of neutrophils and impairs immune tolerance in the last trimester (142, 143)
Recruits Th17 for generating IL-17 to endorse trophoblast proliferation and control the apoptosis in the first trimester (32)	Mediates the alteration of T cells to Th1 and the generation of Th1 cytokines, leading to immune variations in the first trimester (86, 124)	Accumulates excess ROS to disturb the proliferation of trophoblasts in the second trimester (134)	
Produces the bias of Th2 polarization for the toleration of gestation in the first trimester (35, 40)		Attracts circulating monocytes to damage the vascular endothelium in the second trimester (137)	
Enhances the proliferation and vigor of ESC through Akt and MAPK**/**Erk1/2 signaling pathway, for decidualization in the first trimester (93, 94, 97)		Leads to systematic inflammatory disorders in the third trimester (140)	
Regulates the growth and invasion of EVT for deeper implantation in the first trimester (98)			
Prompts the proliferation and anti-apoptosis of DSC in the first trimester (51, 52)			
Rationalizes the M1/M2 ratio for appropriate vascular transformation in the second trimester (109)			
Increases the level of proangiogenic factors for vascular remodeling in the second trimester (114, 115)			
Recruits more macrophages into uterine tissues for an **“**inflammatory microenvironment**”** in the last trimester and the onset of parturition (48)			

## Author contributions

ZL performed the literature research, wrote the manuscript, and prepared the tables. J-LS made great contributions to revising the manuscript and responding to reviewers. MC and Z-MZ revised the manuscript. M-QL and JS designed and wrote the review, supervised, and critically reviewed the complete manuscript. All authors contributed to the article and approved the submitted version.

## Glossary

**Table:**

CCL2	C-C motif ligand 2
MCP-1	monocytic chemotactic protein 1
VSMCs	vascular smooth muscle cells
CCR	C–C chemokine receptor type
ACKR	atypical chemokine receptor
TNF-α	tumor necrosis factor alpha
TAM	tumor-associated macrophages
DRG	dorsal root ganglia
Sarm1	sterile alpha and Toll/interleukin-1 receptor motif-containing1
VCAM	vascular cell adhesion molecule
ICAM	intercellular adhesion molecule
ERK	extracellular signal-regulated kinase
MAPK	mitogen-activated protein kinase
vCTB	villous cytotrophoblast
STB	Syncytiotrophoblast
EVT	extravillous trophoblast
DSC	decidual stromal cell
ESC	endometrium stromal cell
DIC	decidual immune cell
APC	antigen-presenting cell
IL-1β	interleukin-1 beta
PTGS2/COX2	prostaglandin G/H synthase 2
AP-1	activator protein-1
NF-kB	nuclear factor kappa B
ERΑ36	estrogen receptor Α36
TLR	toll-like receptor
hCG	human chorionic gonadotropin
PGF2Α	prostaglandin F2Α
PTGFR	PGF2Α receptor
HUSMC	human uterine smooth muscle cells
PLC	phospholipase C
PKC	protein kinase C
PI3K	phosphatidylinositol-4,5-bisphosphate 3-kinase
VIP	vasoactive intestinal peptide
RANKL/TNFSF11	receptor-activator of NF-kB ligand
c-JNK	c-Jun N-terminal kinase
PAR-1	proteinase-activated receptors-1
MEK	mitogen-activated protein kinase kinase
InsP3	PLC-inositol 1,4,5-trisphosphate
HO-1	Heme oxygenase-1
LPA	lysophosphatidic acid
GPR	G protein-coupled receptor
JAK	Janus kinase
IL-1Ra	IL-1 receptor antagonist
IFN-γ	Interferon Gamma
M-MDSC	Myelomonocytic myeloid-derived suppressor cell
STAT	transducer and activator of transcription
SOCS	Suppressor of Cytokine Signaling
TJP	tight junction protein
COX	cyclooxygenase
Ang	angiopoietin
HUVEC	human umbilical vein endothelial cells
TIE	tyrosine kinase *via* immunoglobulin-like and EGF-like domains
NRP	neuropilin
Ets	E26 transformation-specific
ECM	extracellular matrix
PRL	recurrent pregnancy loss
Nrf2	nuclear factor erythroid 2-related factor
PTL	Preterm labor
IAI	intra-amniotic infection
